# High Dietary Intake of Iron Might Be Harmful to Atrial Fibrillation and Modified by Genetic Diversity: A Prospective Cohort Study

**DOI:** 10.3390/nu16050593

**Published:** 2024-02-22

**Authors:** Zierdi Habudele, Ge Chen, Samantha E. Qian, Michael G. Vaughn, Junguo Zhang, Hualiang Lin

**Affiliations:** 1 Department of Epidemiology, School of Public Health, Sun Yat-Sen University, Guangzhou 510275, China; habudele@mail2.sysu.edu.cn (Z.H.); cheng237@mail2.sysu.edu.cn (G.C.); 2 College of Arts and Sciences, Saint Louis University, St. Louis, MO 63108, USA; sami.qian@slu.edu; 3 School of Social Work, Saint Louis University, St. Louis, MO 63103, USA; michael.vaughn@slu.edu

**Keywords:** atrial fibrillation, iron intake, SNPs

## Abstract

Some studies suggest an association between iron overload and cardiovascular diseases (CVDs). However, the relationship between dietary iron intake and atrial fibrillation (AF) remains uncertain, as does the role of genetic loci on this association. The study involved 179,565 participants from UK Biobank, tracking incident atrial fibrillation (AF) cases. Iron intake was categorized into low, moderate, and high groups based on dietary surveys conducted from 2009 to 2012. The Cox regression model was used to estimate the risk of AF in relation to iron intake, assessing the hazard ratio (HR) and 95% confidence interval (95% CI). It also examined the impact of 165 AF-related and 20 iron-related genetic variants on this association. Pathway enrichment analyses were performed using Metascape and FUMA. During a median follow-up period of 11.6 years, 6693 (3.97%) incident AF cases were recorded. A total of 35,874 (20.0%) participants had high iron intake. High iron intake was associated with increased risk of AF [HR: 1.13 (95% CI: 1.05, 1.22)] in a fully adjusted model. Importantly, there were 83 SNPs (11 iron-related SNPs) that could enhance the observed associations. These genes are mainly involved in cardiac development and cell signal transduction pathways. High dietary iron intake increases the risk of atrial fibrillation, especially when iron intake exceeds 16.95 mg. The association was particularly significant among the 83 SNPs associated with AF and iron, the individuals with these risk genes. Gene enrichment analysis revealed that these genes are significantly involved in cardiac development and cell signal transduction processes.

## 1. Introduction

Atrial fibrillation (AF), one of the most prevalent supraventricular arrhythmias globally, significantly increases the risk of stroke, heart failure, and other cardiovascular diseases (CVDs). These complications not only lead to high medical expenses, but also pose a substantial burden on public health systems [1]. The incidence and prevalence of AF are on the rise globally, according to GBD 2019 data. The estimated number of AF cases is 59.7 million in 2019, which is approximately double the number observed in 2009 [2]. Despite the growing public health challenge due to AF, specific prevention has received little attention.

In addition to aging, several modifiable risk factors for AF have been well established and described, including diet-related risk factors such as hypertension, diabetes, and obesity [3]. However, epidemiological data of specific dietary factors, especially micronutrients, in relation to new-onset AF are limited. Iron is one of the essential micronutrients for the human body. Some studies suggested a connection between iron and atrial fibrillation, while others contradict this finding, leading to conflicting results [3]. Recently, a Mendelian design work indicates a causal relationship between genetically determined higher iron status and an increased risk of atrial fibrillation [4]. The body’s iron stores are derived from dietary sources. When the body’s iron content increases, vital organs like the heart start to absorb iron, and excess iron can lead to myocardial diseases, pericarditis, and arrhythmias [5]. However, no comprehensive evaluation has been performed on the association between dietary iron intake and the risk of atrial fibrillation.

Some studies suggest that iron overload may have a negative impact on the occurrence and progression of atrial fibrillation, and SNPs related to hepcidin genes may participate in the development of atrial fibrillation by affecting iron metabolism and distribution. An MR study has shown a causal relationship between genetically determined higher iron status and increased risk of atrial fibrillation [4]. Furthermore, genetic factors play an important role in the occurrence and development of atrial fibrillation. Research has shown that genetic mutations and polymorphisms are associated with the risk of atrial fibrillation [6,7]. However, there is still controversy over the relationship between iron related SNPs and the occurrence and development of atrial fibrillation, these studies provide a new perspective for us to gain a deeper understanding of the etiology of atrial fibrillation. It needs to comprehensively consider multiple factors such as genetics, environment, and lifestyle to fully reveal the pathogenesis of atrial fibrillation and provide more effective strategies for prevention and treatment.

We used data from the UK Biobank cohort to explore the relationship between dietary iron intake and the risk of AF. We also examined whether the presence of specific genetic variants, comprising 165 SNPs related to AF and 20 SNPs related to iron, influenced the relationship [4,8,9,10,11,12,13,14,15,16]. Furthermore, we examined if genes corresponding to these SNPs provide evidence of a shared enriched pathway.

## 2. Materials and Methods

### 2.1. Study Design and Data Collection

UK Biobank is a large population-based cohort of over 500,000 participants aged 37–73 years. The participants were recruited in 22 assessment centers in England, Wales and Scotland between 2006 and 2010 [17]. The participants completed touchscreen self-report questionnaires, online computer-assisted personal interviews, and physical measurements during the baseline survey [17,18].

**Figure 1:**
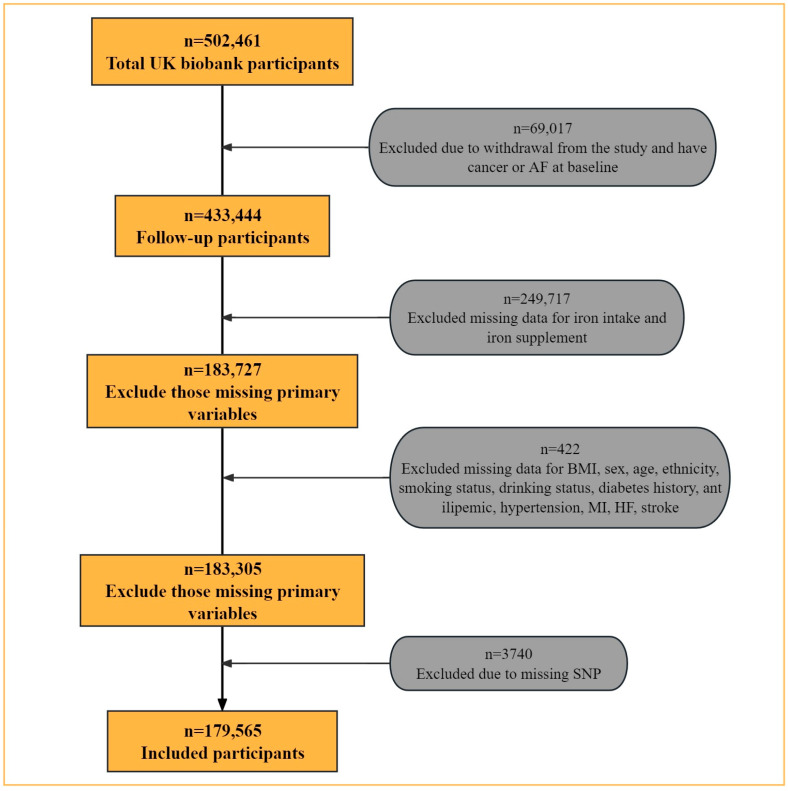
Flow chart for selection of the study participants.

In this study, we excluded 69,017 participants who had cancer or AF at baseline or failed to complete the follow-up. We further excluded 250,139 participants with incomplete or outlier data on dietary iron intake. Finally, we excluded 3740 participants with missing SNP data, yielding a final study population of 179,565 participants (Figure 1).

UKB obtained ethical approval from the North West-Haydock Research Ethics Committee (11/NW/0382) for the protection of subjects and adheres to the principles expressed in the Declaration of Helsinki. All participants provided written informed consent at the start of enrolment.

### 2.2. Dietary Iron Intake Assessment

Participants completed five rounds of 24 h dietary recalls between 2009 and 2012 using Oxford WebQ. Iron intake (mg) was estimated based on yesterday’s food and drink consumption from the dietary questionnaire, excluding any supplements [19,20]. Of 210,959 participants who completed at least one dietary recall, we included 179,565 of them (54.8% women). We categorized the collected iron intake data into three groups of low, moderate, and high intakes using 0–20% (≤10.08 mg), 20–80% (10.08–16.95 mg) and more than 80% (≥16.95 mg) [21]. If participants reported that they had not normally eaten the previous day during a 24 h session (e.g., due to fasting, illness, or other reasons), the dietary data was excluded. Timely data collection would reduce potential recall bias.

### 2.3. Follow-Up and Outcomes

Participants were followed up from the time of the recruitment to one of the following events: death, loss to follow-up, or the end date of follow-up (31 March 2021), whichever came first. The AF diagnosis was obtained from the primary care records, hospital admission records, and death registration. Information on AF was defined by the International Classification of Diseases, 10th Revision (ICD-10) code I48.

### 2.4. Single Nucleotide Polymorphism Selection and Genotyping

A large GWAS meta-analysis showed that the 165 variants constituted the optimal list of variants for the current polygenic risk score of AF (Table S1) [9]. Candidate genes linked to AF using four criteria: (1) genes with a protein-altering variant or in strong linkage disequilibrium (r^2^ > 0.80); (2) genes whose expression levels are significantly associated with AF variants, as per GTEx data (*p* < 1.14 × 10^−9^); (3) genes significantly enriched in DEPICT analysis (FDR < 0.05); and (4) genes nearest to the index variant in their respective loci [11]. A total of 20 iron-related SNPs and their corresponding genes were obtained through a literature search (Table S2) [4,8,10,11,12,13,14,15,16]. If there are no corresponding genes, we executed a query and supplement from NCBI. Based on the number of SNP alleles, each SNP was classified into two groups (no alleles vs. at least one allele).

### 2.5. GO and Pathway Enrichment Analysis

Metascape and Fuma were used to analyze the pathway of the gene ontology corresponding to 83 SNPs. Metascape (https://metascape.org/, accessed on 1 January 2024) [22] was used to analyze the enrichment of 62 genes corresponding to 83 SNPs. The enrichment factor is regarded as the ratio of the observed counts to the counts expected by chance). Terms with an enrichment factor > 1.5, a minimum count of 3, and a *p*-values < 0.01, were grouped into clusters according to the similarity of their membership. 

Functional Mapping and Annotation (FUMA, https://fuma.ctglab.nl/, accessed on 1 January 2024) is an online tool that integrates information from state-of-the-art biological data sources into a single platform for functional annotation, visualization, and interpretation of the results for genetic association studies, and for rapid insight into the directional biological significance of important genetic associations [23]. GENE2FUNC (DEPT. Complex Trait Genetics at Vu University Amsterdam),a tool of FUMA, provides information on the expression of priority genes and detects the enrichment of gene sets in the pathway [24]. The genes listed in Table S4 served as inputs for this analysis.

### 2.6. Covariates

Demographic information was self-reported, including sex (female, male); age (≤65 years, >65 years); and ethnicity (White, nonwhite). Both smoking status (never, previous, current) and alcohol consumption (never, previous, current) were collected via a touch-screen self-report questionnaire. PA (low, moderate and high) contains derived MET (Metabolic Equivalent Task) scores data based on IPAQ (International Physical Activity Questionnaire) guidelines. Medical history was obtained from primary care data, including myocardial infarction (filed ID 131299/131301/131303, ICD code I21–I23, and diagnoses—ICD10 code I241 I252), heart failure (filed ID 131355, ICD code I50, and diagnoses—ICD10 code I110), stroke (filed ID131361/131363/131365/131367/131369, ICD code I60–I64), hypertension (field ID 131287/131295, ICD code I10/I15 and self-reported), and diabetes (field ID 130707/130709/130711/130713/130715, ICD code E10–E14) [22]. Baseline CVD means that at least one of myocardial infarction, heart failure, or stroke is present. Drug use, including history of use of antilipemics and antidiabetics. Antidiabetics include metformin, insulin, gliclazide, pioglitazone, rosiglitazone, glimepiride, glyburide, glipizide, repaglinide, tolbutamide, acarbose, nateglinide; antilipemics include simvastatin, atorvastatin, rosuvastatin, pravastatin, fluvastatin. Body mass index (BMI) (kg/m^2^) (obese (BMI ≥ 30 kg/m^2^), overweight (25 ≤ BMI < 30 kg/m^2^), normal (18.5 ≤ BMI < 25 kg/m^2^), underweight (BMI < 18.5 kg/m^2^)) was computed by weight in kilograms divided by height in meters squared [25,26]. Iron supplementation (yes/no) was calculated based on the average of the number of surveys evaluated. Iron intake was extracted from dietary foods for modelling analysis.

### 2.7. Statistical Analysis

Differences in baseline characteristics across iron intake groups were expressed by an χ^2^ test for categorical covariates and by ANOVA for continuous covariates. Cox proportional hazards models were carried out to estimate the hazard ratios (HRs) and 95% confidence interval (CI) for the associations between iron intake and AF occurrence. Additionally, the models examined this relationship within the context of 185 SNPs.

The group with the lowest iron intake was designated as the reference group for the Cox proportional hazards regression model. We first performed a univariate model to explore the relationship between iron intake and risk of atrial fibrillation. A multivariate model was further conducted with adjusting for age, sex, ethnicity, smoking status, drinking status, BMI, diabetes history, antilipemic, iron supplementation, hypertension, myocardial infarction, heart failure, and stroke.

All statistical analyses were conducted using the R software (version 4.3.2).

### 2.8. Subgroup Analysis

We conducted a subgroup analysis to examine the relationship between iron intake and the incidence of AF by sex, age, and ethnicity.

## 3. Results

### 3.1. Baseline Characteristics

A total of 179,565 participants were included in the analyses with low, moderate, and high iron intake groups of 35,967 (20.0%), 107,724 (60.0%), and 35,874 (20.0%), respectively. Participants older than 65 years totaled 14.4% (*n* = 25,899), 45.2% of the participants (*n* = 81,136) were male, and 3.7% (*n* = 6693) had atrial fibrillation. The differences of iron intake groups in baseline characteristics are shown in Table 1. The high iron intake group was more likely to be aged ≤65 years, male, overweight, and current alcohol drinkers. Additionally, they were less likely to use iron supplements and had a lower prevalence of hypertension and smoke less.

### 3.2. Dietary Iron Intake and the Risk of Incident Atrial Fibrillation

During a median follow-up of 11.1 (interquartile range 10.3–11.8) years, 1179 (3.4%), 3943 (3.7%), and 1573 (4.4%) AF events occurred in the low, moderate, and high iron intake groups, respectively. Using the low iron intake group as a reference, the unadjusted HRs for the medium and high iron intake groups were 1.11 (95% CI: 1.04, 1.18) and 1.33 (95% CI: 1.24, 1.44), and adjusted HRs were 1.05 (95% CI: 0.98, 1.12) and 1.13 (95% CI: 1.05, 1.22) as presented in Table 2.

### 3.3. Incident Atrial Fibrillation according to Different SNPs and Dietary Iron Intake

The analyzed SNPs are presented in Table S3. There were 83 SNPs (11 iron-related SNPs) that promoted the occurrence of AF under high iron intake only when risk genes were present. A total of 65 SNPs found the corresponding genes. When considering only the risk genes, all 13 SNPs were associated with promoting the occurrence of AF through the intake of moderate and high levels of iron. These SNPs included rs11264280, rs12188351, rs17380837, rs2283038, rs34969716, rs67249485, rs73041705, rs79187193, rs9506925, rs2413450, rs4820268, rs5756506, and rs855791. Among them, there were 4 SNPs related to iron, namely rs4820268, rs2413450, rs5756506, and rs855791.

### 3.4. Enrichment of Input Genes in Gene Sets

**Figure 2:**
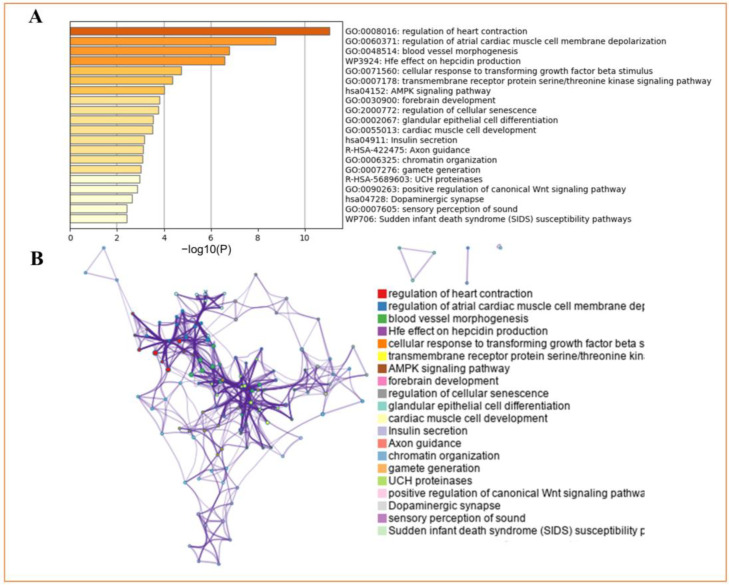
Functional enrichment of differential genes in Metascape. (**A**) GO-KEGG enrichment analysis of differential genes. (**B**) A cluster network of enriched pathways, where nodes sharing the same cluster are often positioned close to each other.

GO enrichment analyses of genes corresponding to the 83 SNPs were performed using metascape. The results showed that these genes were enriched in the regulation of heart contraction (GO:0008016), regulation of atrial cardiac muscle cell membrane depolarization (GO:0060371), blood vessel morphogenesis (GO:0048514), Hfe effect on hepcidin production (WP3924), and cellular response to transforming growth factor beta stimulus (GO:0071560) (Figure 2 and Table 3).

**Figure 3:**
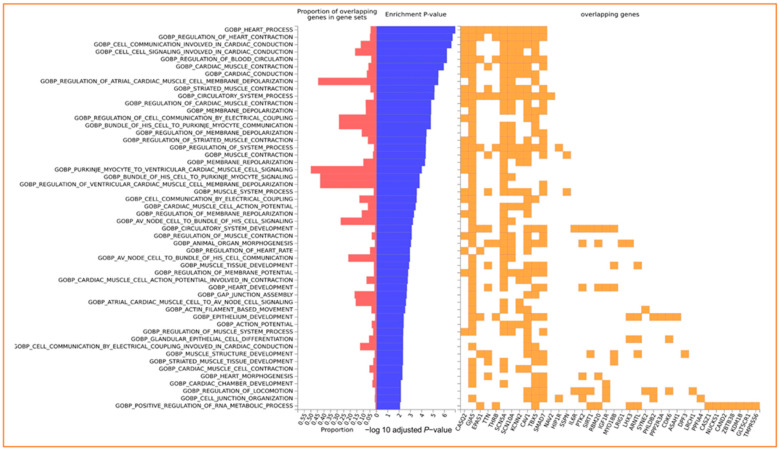
Enrichment of input genes in Gene Sets in FUMA. The −log10 adjusted *p*-value reflects the statistical significance of enrichment, taking into account multiple testing corrections using the Benjamini–Hochberg (BH) method. The BH method controls the false discovery rate, mitigating the likelihood of obtaining false positives when testing multiple gene sets for enrichment.

Simultaneous gene enrichment using FUMA resulted in, among others, heart process, regulation of contraction, cell communication involved in cardiac conduction, cell signaling involved in cardiac conduction, and regulation of blood circulation pathways (Figure 3 and Table S4).

The enrichment analyses of the corresponding genes consistently unveiled the biological processes closely associated with cardiac development and cardiac cell signal transduction.

### 3.5. Subgroup Analysis

The results from subgroup analyses are presented in Figure S1. The associations between iron intake and AF were stronger in young, male, and white participants (*p* < 0.05).

## 4. Discussion

Employing the UK Biobank cohort, our objective was to provide a comprehensive evaluation of the relationship between dietary iron intake and atrial fibrillation. Three key findings are indicated by our analysis. First, high iron intake was associated with a 13% increase in the incidence of AF after adjusting for important covariates. Second, an increased iron intake was associated with an elevated occurrence of AF among risk gene carriers of the 83 SNPs. Third, the enrichment analyses of SNP-associated genes consistently converge on the biological processes of cardiac development and myocardial cell signal transduction, further validating our hypothesis.

Observational studies have linked iron status to both cardiac arrhythmias and atrial fibrillation [27]. Previous studies have found that the amount of iron in the body is closely related to the occurrence of AF. For example, epidemiological investigations have revealed that patient groups with elevated ferritin or serum iron levels tend to have a higher incidence of AF [28]. These observational studies provide valuable clues to the association between iron and atrial fibrillation. Although previous studies have examined the relationship between dietary iron intake and cardiovascular diseases, their conclusions have been inconsistent. Part of the reason may be that these studies often relied on single dietary questionnaires, which can be challenging to accurately reflect an individual’s long-term iron intake [29]. Alternatively, due to the small sample size or observational nature of the study population, it may be difficult to draw statistically significant conclusions. In addition, there is relatively little research specifically focused on the relationship between iron intake and atrial fibrillation, which further increases the uncertainty in this field.

Researchers such as Yang have further pointed out the potential mechanism of iron induced atrial fibrillation through in-depth experiments and theoretical analysis. They found that excessive iron can increase the risk of atrial fibrillation through oxidative stress, affecting the electrophysiological properties of heart cells, or promoting inflammatory responses [30]. These findings offer significant theoretical insights into understanding the relationship between iron and AF. It is worth noting that iron excretion in the human body is relatively limited, making iron homeostasis largely dependent on the regulation of iron absorption. Iron absorption, in turn, is influenced by various dietary factors (such as the form of iron and the overall composition of the diet) as well as the body’s iron status [31]. This complex regulatory mechanism adds another layer of complexity to the already elusive relationship between iron and AF. Furthermore, the enrichment analysis of SNP-associated genes consistently highlights the biological processes, including cardiac development and myocardial cell signal transduction. This convergence supports the notion that the genetic variants are intricately linked to key aspects of cardiac physiology. The enrichment results add a layer of biological context to the observed association between iron intake, genetic predisposition, and atrial fibrillation.

We wanted to further explore the underlying mechanisms or possible reasons for the increased risk of AF in SNPs with risk genes present. Rs1799945 in the HFE gene and rs855791 in the TMPRSS6 gene exhibited associations with all four iron biomarkers at genome-wide significance (*p* < 5 × 10^−8^) [32]. TMPRSS6 was related to serum iron concentration and was directly involved in the regulation of dietary iron absorption and use [10]. Tanaka et.al found that 20–30% of the variation in iron concentration was attributed to the reduced activity of the Matriptase-2 protein. The latter was determined by environmental factors, including dietary iron intake. At the same time, each TMPRSS6 variant explained only about 1% of the variation in iron concentration [33]. Regarding the research on TMPRSS6 and CVDs, Nauffal et al., showed that it may be related to myocardial interstitial fibrosis [34]. Research shows that rs651007 and rs411988 (TEX14, LOC105371842 gene) affect ferritin concentration, and the former also reduces the risk of atherosclerosis [12]. Rs179945 (HEF gene) is associated with hypertension [35]. High blood pressure is a risk factor for atrial fibrillation. There are currently no studies on the relationship between rs2245321 (gene LINC01378) and CVD. Rrs6486121 (gene ARNTL) is significantly associated with intestinal cholesterol absorption [36]. It may further affect the metabolism and absorption of iron in the intestine. Among the iron-related SNPs, including SNPs rs4921915, rs6486121 (gene ARNTL), rs1799945 (genes HFE, HFE-AS1), rs744653, rs651007, rs85579, and rs411988, it was observed that rs411988 (TEX14, LOC105371842) is associated with ferritin. Notably, the ratio of ferritin to transferrin/iron is causally related to atherosclerosis [12].

The strengths of this study include its population-based design, prospective collection of lifestyle data, the use of validated food frequency questionnaires, large sample sizes and long-term follow-up, and multivariate-adjusted models to account for the effects of confounders. There are also some limitations to our study. The 24 h dietary recall information is also based on a single assessment at baseline, and eating habits may have changed during the follow-up period and lead to the underestimation of the association between intake and cognition. However, any such misclassification in the assessment of exposure may be undifferentiated among study participants and may attenuate the risk estimate associated with the outcome to zero. Still, we were able to see differential associations with the incidence of atrial fibrillation in our study. Although we controlled for some disease history and medication, the presence of subclinical disease may lead to changes in dietary habits, and there is still the possibility of residual confounding of the association between iron intake and the development of atrial fibrillation. In the end, we are only doing initial exploration, and future studies can go further and cross-validate the current results in different predictive models or different populations.

## 5. Conclusions

High dietary iron intake increases the risk of atrial fibrillation, especially when iron intake exceeds 16.95 mg. The association was particularly significant among the 83 SNPs associated with AF and iron, the individuals with these risk genes. Gene enrichment analysis revealed these genes are significantly involved in cardiac development and cell signal transduction processes. Strict control of iron intake, avoidance of excessive supplementation, and limiting iron-rich foods are key for those with iron overload or a high AF risk.

## Figures and Tables

**Table 1:** Baseline characteristics of the study participants stratified by dietary iron intake.

Characteristics	Overall (*n* = 179,565)	Low (*n* = 35,967)	Moderate (*n* = 107,724)	High (*n* = 35,874)	*p*-Value
Atrial fibrillation (%)					
No	172,872 (96.3)	34,788 (96.7)	103,781(96.3)	34,303 (95.6)	<0.001
Yes	6693 (3.7)	1179 (3.3)	3943 (3.7)	1571 (4.4)	
Sex (%)					
Female	98,429 (54.8)	23,019 (64.0)	60,741 (56.4)	14,669 (40.9)	<0.001
Male	81,136 (45.2)	12,948 (36.0)	46,983 (43.6)	21,205 (59.1)	
Age (%)					
≤65 years	153,666 (85.6)	31,579 (87.8)	91,902 (85.3)	30,185 (84.1)	<0.001
>65 years	25,899 (14.4)	4388 (12.2)	15,822 (14.7)	5689 (15.9)	
Ethnicity (%)					
Nonwhite	7958 (4.4)	2885 (8.0)	3754 (3.5)	1319 (3.7)	<0.001
White	171,607 (95.6)	33,082 (92.0)	103,970 (96.5)	34,555 (96.3)	
Setting (%)					
Urban	150,958 (84.9)	30,870 (86.8)	90,099 (84.4)	29,989 (84.5)	<0.001
Rural	26,787 (15.1)	4676 (13.2)	16,606 (15.6)	5505 (15.5)	
BMI (%)					
Normal	66,637 (37.1)	12,143 (33.8)	41,050 (38.1)	13,444 (37.5)	<0.001
Underweight	960 (0.5)	200 (0.6)	552 (0.5)	208 (0.6)	
Overweight	74,597 (41.5)	14,716 (40.9)	44,632 (41.4)	15,249 (42.5)	
Obese	37,371 (20.8)	8908 (24.8)	21,490 (19.9)	6973 (19.4)	
Iron supplement (%)					
No	173,391 (96.6)	34,600 (96.2)	104,192 (96.7)	34,599 (96.4)	<0.001
Yes	6174 (3.4)	1367 (3.8)	3532 (3.3)	1275 (3.6)	
TPI (mean (SD))	−1.57 (2.88)	−1.22 (3.04)	−1.68 (2.82)	−1.59 (2.87)	<0.001
Hypertension (%)					
No	148,867 (82.9)	29,703 (82.6)	89,697 (83.3)	29,467 (82.1)	<0.001
Yes	30,698 (17.1)	6264 (17.4)	18,027 (16.7)	6407 (17.9)	
Smoking status (%)					
Never	102,830 (57.3)	20,464 (56.9)	62,395 (57.9)	19,971 (55.7)	<0.001
Previous	62,569 (34.8)	11,570 (32.2)	37,702 (35.0)	13,297 (37.1)	
Current	14,166 (7.9)	3933 (10.9)	7627 (7.1)	2606 (7.3)	
Drinking status (%)					
Never	5814 (3.2)	1834 (5.1)	3082 (2.9)	898 (2.5)	<0.001
Previous	5311 (3.0)	1492 (4.1)	2880 (2.7)	939 (2.6)	
Current	168,440 (93.8)	32,641 (90.8)	101,762 (94.5)	34,037 (94.9)	
PA (%)					
Low	27,931 (18.3)	6493 (21.9)	16,701 (18.2)	4737 (15.1)	<0.001
Moderate	64,486 (42.3)	12,269 (41.3)	39,392 (43.0)	12,825 (40.9)	
High	60,134 (39.4)	10,917 (36.8)	35,437 (38.7)	13,780 (44.0)	
History of diabetes (%)					
No	172,522 (96.1)	34,396 (95.6)	103,712 (96.3)	34,414 (95.9)	<0.001
Yes	7043 (3.9)	1571 (4.4)	4012 (3.7)	1460 (4.1)	
History of obesity (%)					
No	175,658 (97.8)	35,026 (97.4)	105,491 (97.9)	35,141 (98.0)	<0.001
Yes	3907 (2.2)	941 (2.6)	2233 (2.1)	733 (2.0)	
Antidiabetics (%)					
No	174,519 (97.2)	34,798 (96.7)	104,878 (97.4)	34,843 (97.1)	<0.001
Yes	5046 (2.8)	1169 (3.3)	2846 (2.6)	1031 (2.9)	
Antilipemic (%)					
No	155,360 (86.5)	31,104 (86.5)	93,481 (86.8)	30,775 (85.8)	<0.001
Yes	24,205 (13.5)	4863 (13.5)	14,243 (13.2)	5099 (14.2)	
Baseline CVD					
No	172,998 (96.3)	34,623 (96.3)	103,937 (96.5)	34,438 (96.0)	<0.001
Yes	6567 (3.7)	1344 (3.7)	3787 (3.5)	1436 (4.0)	

Note: Categorical data presented as count (%), continuous data presented as mean ± SD. BMI, body mass index (kg/m^2^) (obese (BMI ≥ 30 kg/m^2^), overweight (25 ≤ BMI < 30 kg/m^2^), normal (18.5 ≤ BMI < 25 kg/m^2^), underweight (BMI < 18.5 kg/m^2^)); TPI, Townsend deprivation index; PA, physical activity; CVD, includes myocardial infarction (MI), heart failure (HF) and stroke, comorbidities refer to at least one type of CVD; antidiabetics include metformin, insulin, gliclazide, pioglitazone, rosiglitazone, glimepiride, glyburide, glipizide, repaglinide, tolbutamide, acarbose, nateglinide; antilipemics include simvastatin, atorvastatin, rosuvastatin, pravastatin, fluvastatin.

**Table 2:** Risk of atrial fibrillation with dietary iron intake.

	Events *n* (%)	HR (95% CI)	*p*-Value	Adjusted HR (95% CI) ^a^	*p*-Value
Low iron intake (*n* = 35,967)	1179 (3.28)	Reference		Reference	
Moderate iron intake (*n* = 107,724)	3943 (3.66)	1.11 (1.04, 1.18)	<0.001	1.05 (0.98, 1.12)	0.18
High iron intake (*n* = 35,874)	1571 (4.38)	1.33 (1.24, 1.44)	<0.001	1.13 (1.05, 1.22)	<0.001

HR, hazard ratio; CI, confidence interval. ^a^ Adjusted for age, sex, ethnicity, smoking, drinking, BMI, diabetes history, antilipemic, iron supplement, hypertension, myocardial infarction, heart failure, stroke.

**Table 3:** Top 20 clusters with their representative enriched terms (one per cluster) in Metascape.

GO	Category	Description	Count	%	Log10 (*p*)	Log10 (*q*)
GO:0008016	GO Biological Processes	regulation of heart contraction	10	16.67	−11.06	−6.72
GO:0060371	GO Biological Processes	regulation of atrial cardiac muscle cell membrane depolarization	4	6.67	−8.76	−5.13
GO:0048514	GO Biological Processes	blood vessel morphogenesis	9	15	−6.79	−3.59
WP3924	WikiPathways	Hfe effect on hepcidin production	3	5	−6.59	−3.45
GO:0071560	GO Biological Processes	cellular response to transforming growth factor beta stimulus	5	8.33	−4.74	−1.97
GO:0007178	GO Biological Processes	transmembrane receptor protein serine/threonine kinase signaling pathway	5	8.33	−4.36	−1.65
hsa04152	KEGG Pathway	AMPK signaling pathway	4	6.67	−4	−1.44
GO:0030900	GO Biological Processes	forebrain development	6	10	−3.82	−1.3
GO:2000772	GO Biological Processes	regulation of cellular senescence	3	5	−3.77	−1.26
GO:0002067	GO Biological Processes	glandular epithelial cell differentiation	3	5	−3.53	−1.08
GO:0055013	GO Biological Processes	cardiac muscle cell development	3	5	−3.51	−1.06
hsa04911	KEGG Pathway	Insulin secretion	3	5	−3.17	−0.83
R-HAS-422475	Reactome Gene Sets	Axon guidance	6	10	−3.11	−0.79
GO:0006325	GO Biological Processes	chromatin organization	7	11.67	−3.09	−0.79
GO:0007276	GO Biological Processes	gamete generation	7	11.67	−3.01	−0.74
R-HSA-5689603	Reactome Gene Sets	UCH proteinases	3	5	−2.96	−0.72
GO:0090263	GO Biological Processes	positive regulation of canonical Wnt signaling pathway	3	5	−2.86	−0.67
hsa04728	KEGG Pathway	Dopaminergic synapse	3	5	−2.64	−0.51
GO:0007605	GO Biological Processes	sensory perception of sound	3	5	−2.42	−0.36
WP706	WikiPathways	Sudden infant death syndrome (SIDS) susceptibility pathways	3	5	−2.4	−0.36

## Data Availability

Publicly available datasets were analyzed in this study. This data can be found here: https://www.ukbiobank.ac.uk/ (accessed on 1 October 2023).

## Supplementary Materials

The following supporting information can be downloaded at: https://www.mdpi.com/article/10.3390/nu16050593/s1, Table S1: Atrial fibrillation-associated 165 SNPs; Table S2: Iron-related 20 SNPs; Table S3: Association results for the 185 single nucleotide polymorphisms analyzed and the distribution of Atrial fibrillation; Table S4: Total number of enrichments of the input genes in FUMA; Figure S1: Subgroup analysis.
