# Supplementary material for: High Dietary Intake of Iron Might Be Harmful to Atrial Fibrillation and Modified by Genetic Diversity: A Prospective Cohort Study
Source: Nutrients. 2024 Feb 22;16(5):593. doi: 10.3390/nu16050593 (PMC10935150; doi:10.3390/nu16050593)
Supplement: Supplementary file 1 [file nutrients-16-00593-s001.zip › nutrients-2830307-supplementary.pdf]

Supplement Material

Table S1. Atrial fibrillation-associated 165 SNPs.

Rs ID	Position	Risk/Reference Allele	Effect (beta)	StdErr	*p*-Value
rs284277	chr1:10790797	C/A	0.0422	0.0069	1.25×10^{-9}
rs7529220	chr1:22282619	C/T	0.0621	0.0098	1.98×10^{-10}
rs2885697	chr1:41544279	G/T	0.0439	0.007	2.88×10^{-10}
rs11590635	chr1:49309764	A/G	0.1456	0.0248	4.12×10^{-9}
rs146518726	chr1:51535039	A/G	0.1605	0.0207	8.27×10^{-15}
rs1545300	chr1:112464004	C/T	0.0558	0.0073	1.48×10^{-14}
rs4073778	chr1:116297758	A/C	0.0486	0.0067	4.96×10^{-13}
rs10465885	chr1:147232740	C/T	0.0302	0.0067	5.74×10^{-6}
rs79187193	chr1:147255831	G/A	0.1162	0.0153	3.15×10^{-14}
rs6689306	chr1:154395946	A/G	0.046	0.0068	1.36×10^{-11}
rs4999127	chr1:154714006	A/G	0.0827	0.0098	4.28×10^{-17}

rs11264280	chr1:154862952	T/C	0.1347	0.0071	3.07×10^{-79}
rs72700114	chr1:170193825	C/G	0.2021	0.013	3.29×10^{-54}
rs72700118	chr1:170194823	A/C	0.1227	0.0101	9.52×10^{-34}
rs577676	chr1:170587340	C/T	0.0923	0.0067	1.62×10^{-43}
rs10753933	chr1:203026214	T/G	0.0609	0.0067	9.84×10^{-20}
rs4951258	chr1:205691316	A/G	0.0376	0.0067	2.10×10^{-8}
rs7578393	chr2:26165528	T/C	0.0614	0.0088	2.42×10^{-12}
rs11689011	chr2:46541176	T/C	0.0321	0.0103	3.12×10^{-8}
rs11125871	chr2:61470126	C/T	0.0394	0.0068	6.42×10^{-9}
rs2540949	chr2:65284231	A/T	0.0659	0.0068	2.95×10^{-22}
rs6747542	chr2:70106832	T/C	0.0554	0.0067	1.10×10^{-16}
rs72926475	chr2:86594487	G/A	0.0683	0.0102	2.37×10^{-11}
rs28387148	chr2:127433465	T/C	0.0741	0.0113	6.25×10^{-11}
rs67969609	chr2:145760353	G/C	0.0711	0.0126	1.71×10^{-8}

rs56181519	chr2:175555714	C/T	0.0662	0.0077	6.46×10^{-18}
rs2288327	chr2:179411665	G/A	0.0919	0.0089	7.26×10^{-25}
rs3820888	chr2:201180023	C/T	0.0684	0.0068	5.75×10^{-24}
rs35544454	chr2:213266003	A/T	0.0589	0.0087	1.10×10^{-11}
rs7650482	chr3:12841804	G/A	0.0711	0.007	1.79×10^{-24}
rs73041705	chr3:24463235	T/C	0.0443	0.0073	1.55×10^{-9}
rs7374540	chr3:38634142	A/C	0.0325	0.0068	1.68×10^{-6}
rs7373065	chr3:38710315	T/C	0.2024	0.0251	7.58×10^{-16}
rs6790396	chr3:38771925	G/C	0.0627	0.0068	2.40×10^{-20}
rs34080181	chr3:66454191	G/A	0.0446	0.0069	1.28×10^{-10}
rs17005647	chr3:69406181	T/C	0.0413	0.0069	2.70×10^{-9}
rs6771054	chr3:89489529	T/C	0.0457	0.0068	2.42×10^{-11}
rs10804493	chr3:111554426	A/G	0.0558	0.007	1.63×10^{-15}
rs1278493	chr3:135814009	G/A	0.0389	0.0068	8.77×10^{-9}

rs13077048	chr3:141106954	T/A	0.0493	0.0104	4.75×10^{-10}
rs62274627	chr3:148702947	A/G	0.0297	0.0109	2.46×10^{-8}
rs7612445	chr3:179172979	T/G	0.0493	0.0084	4.81×10^{-9}
rs60902112	chr3:194800853	T/C	0.0445	0.0079	1.72×10^{-8}
rs34104130	chr4:10101300	G/T	0.0365	0.0112	2.55×10^{-11}
rs1458038	chr4:81164723	T/C	0.0434	0.0072	1.74×10^{-9}
rs6841049	chr4:83910712	T/G	0.0242	0.0103	1.95×10^{-8}
rs10006327	chr4:103890980	C/T	0.0364	0.0067	4.42×10^{-8}
rs244017	chr4:111255917	T/G	0.0194	0.0085	0.02247
rs61501369	chr4:111524629	T/C	0.1038	0.008	4.69×10^{-38}
rs6850025	chr4:111596360	A/G	0.1815	0.0154	4.93×10^{-32}
rs67249485	chr4:111699685	T/A	0.3655	0.0081	7.32×10^{-443}
rs3853445	chr4:111761487	T/C	0.1693	0.0076	3.60×10^{-109}
rs79399769	chr4:111925656	C/T	0.1197	0.0232	2.59×10^{-7}

rs1532170	chr4:112165212	G/A	0.0306	0.007	1.26×10^{-5}
rs138311480	chr4:112454295	C/T	0.0739	0.0284	0.00928
rs114904067	chr4:112604821	G/A	0.082	0.0215	0.000136
rs7687819	chr4:113329345	A/G	0.0218	0.0079	0.00612
rs6829664	chr4:114448656	G/A	0.0556	0.0076	1.92×10^{-13}
rs10213171	chr4:148937537	G/C	0.091	0.0134	1.32×10^{-11}
rs10520260	chr4:174447349	A/G	0.0457	0.0073	3.36×10^{-10}
rs12648245	chr4:174641184	T/C	0.0926	0.0127	3.45×10^{-13}
rs6596717	chr5:106427609	C/A	0.0404	0.0068	3.00×10^{-9}
rs337705	chr5:113737062	G/T	0.0564	0.0068	1.63×10^{-16}
rs2012809	chr5:128190363	G/A	0.0582	0.0094	4.92×10^{-10}
rs2040862	chr5:137419989	T/C	0.1084	0.0087	1.08×10^{-35}
rs17118812	chr5:139703286	C/T	0.0431	0.0114	1.83×10^{-10}
rs6580277	chr5:142818123	G/A	0.067	0.0079	1.64×10^{-17}

rs12188351	chr5:168386089	A/G	0.0865	0.0145	2.52×10^{-9}
rs6891790	chr5:172670745	G/T	0.0729	0.0076	4.53×10^{-22}
rs28439930	chr5:173393111	G/C	0.0458	0.0068	1.19×10^{-11}
rs73366713	chr6:16415751	G/A	0.1035	0.0099	1.53×10^{-25}
rs34969716	chr6:18210109	A/G	0.0702	0.0078	1.60×10^{-19}
rs2308655	chr6:31322303	C/G	0.0494	0.012	8.09×10^{-9}
rs3176326	chr6:36647289	G/A	0.0626	0.0085	1.42×10^{-13}
rs12211255	chr6:76188330	A/C	0.057	0.0159	2.60×10^{-10}
rs2031522	chr6:87821501	A/G	0.0436	0.0068	1.47×10^{-10}
rs3951016	chr6:118559658	A/T	0.0648	0.0067	2.15×10^{-22}
rs9401451	chr6:122099152	G/A	0.0733	0.011	2.51×10^{-11}
rs13195459	chr6:122403559	G/A	0.0623	0.007	4.15×10^{-19}
rs4896104	chr6:135119089	C/T	0.0421	0.0103	8.62×10^{-10}
rs117984853	chr6:149399100	T/G	0.1228	0.012	1.34×10^{-24}

rs12700233	chr7:904757	T/G	0.0367	0.0104	5.20×10^{-11}
rs55734480	chr7:14372009	A/G	0.0548	0.0078	2.20×10^{-12}
rs6462079	chr7:28415827	A/G	0.0466	0.0076	8.79×10^{-10}
rs35005436	chr7:74134911	C/T	0.0612	0.0097	3.34×10^{-10}
rs56201652	chr7:92278116	G/A	0.0531	0.0075	1.74×10^{-12}
rs2283038	chr7:106835410	T/C	0.0266	0.012	9.76×10^{-10}
rs11773845	chr7:116191301	A/C	0.1054	0.0067	2.39×10^{-55}
rs55985730	chr7:128417044	G/T	0.0867	0.0149	5.24×10^{-9}
rs7789146	chr7:150661409	G/A	0.0584	0.0087	2.12×10^{-11}
rs35620480	chr8:11499908	C/A	0.054	0.0092	5.15×10^{-9}
rs7508	chr8:17913970	A/G	0.0711	0.0075	1.69×10^{-21}
rs7834729	chr8:21821778	G/T	0.0653	0.0104	3.55×10^{-10}
rs17430364	chr8:118863445	T/A	0.0425	0.0133	4.43×10^{-8}
rs62521286	chr8:124551975	G/A	0.1202	0.0135	4.50×10^{-19}

rs4871397	chr8:124635197	G/C	0.0756	0.0138	4.65×10^{-8}
rs35006907	chr8:125859817	A/C	0.0083	0.0107	2.66×10^{-8}
rs72721963	chr8:135798224	G/A	0.039	0.0174	1.91×10^{-9}
rs6994744	chr8:141740868	C/A	0.0405	0.0066	1.10×10^{-9}
rs10821415	chr9:97713459	A/C	0.0821	0.0067	2.92×10^{-34}
rs4743034	chr9:109632353	A/G	0.0246	0.0122	6.14×10^{-9}
rs10760361	chr9:127178266	G/T	0.0259	0.0106	6.04×10^{-10}
rs2274115	chr9:139094773	G/A	0.0487	0.0076	1.69×10^{-10}
rs12245149	chr10:65321147	C/A	0.047	0.0067	1.66×10^{-12}
rs7096385	chr10:69664881	T/C	0.0707	0.013	4.87×10^{-8}
rs60212594	chr10:75414344	G/C	0.1176	0.0096	9.20×10^{-35}
rs10458660	chr10:77936576	G/A	0.0537	0.0087	6.78×10^{-10}
rs55693294	chr10:105277474	T/C	0.0546	0.0146	0.000182
rs11598047	chr10:105342672	G/A	0.1537	0.009	8.95×10^{-66}

rs35176054	chr10:105480387	A/T	0.1391	0.01	3.21×10^{-44}
rs10749053	chr10:112576695	T/C	0.0555	0.0097	1.05×10^{-8}
rs10741807	chr11:20011445	T/C	0.0729	0.0079	1.59×10^{-20}
rs565449	chr11:95092398	G/A	0.0409	0.0109	2.24×10^{-9}
rs4935786	chr11:121661507	T/A	0.0463	0.0079	4.85×10^{-9}
rs76097649	chr11:128764570	A/G	0.1151	0.0124	1.26×10^{-20}
rs2291437	chr12:24715048	G/T	0.0955	0.0104	5.05×10^{-20}
rs4963776	chr12:24779491	G/T	0.0913	0.0088	1.84×10^{-25}
rs17380837	chr12:26345526	C/T	0.0501	0.0072	4.80×10^{-12}
rs12809354	chr12:32978437	C/T	0.0718	0.0094	2.89×10^{-14}
rs11614818	chr12:56055815	C/T	0.0329	0.007	2.44×10^{-6}
rs2860482	chr12:57105938	A/C	0.054	0.0076	1.21×10^{-12}
rs71454237	chr12:70013415	G/A	0.062	0.0084	1.78×10^{-13}
rs775498	chr12:70071513	G/A	0.0423	0.0074	1.05×10^{-8}

rs12426679	chr12:76237987	C/T	0.0391	0.0067	4.95×10^{-9}
rs883079	chr12:114793240	T/C	0.0981	0.0074	2.84×10^{-40}
rs116904997	chr12:120668534	G/A	0.1116	0.0405	1.28×10^{-8}
rs10773657	chr12:123327900	C/A	0.0575	0.0103	2.54×10^{-8}
rs7134121	chr12:124447346	T/C	0.0317	0.0108	2.00×10^{-11}
rs6560886	chr12:133150210	C/T	0.051	0.009	1.49×10^{-8}
rs9506925	chr13:23368943	T/C	0.0449	0.0075	2.72×10^{-9}
rs1980728	chr13:47247985	G/T	0.0361	0.0116	2.08×10^{-9}
rs35569628	chr13:113872712	T/C	0.0452	0.008	1.38×10^{-8}
rs422068	chr14:23864804	C/T	0.0439	0.007	3.87×10^{-10}
rs1957021	chr14:32924505	C/T	0.0583	0.008	2.27×10^{-13}
rs11156751	chr14:32990437	C/T	0.0719	0.0077	6.94×10^{-21}
rs73241997	chr14:35173775	T/C	0.0733	0.0093	2.94×10^{-15}
rs2738413	chr14:64679960	A/G	0.0778	0.0067	2.55×10^{-31}

rs74884082	chr14:73249419	C/T	0.0493	0.0078	3.48×10^{-10}
rs10873298	chr14:77426525	C/T	0.0401	0.0069	7.07×10^{-9}
rs147301839	chr15:57924714	C/A	0.3328	0.0523	1.93×10^{-10}
rs7170477	chr15:64103777	A/G	0.0393	0.0072	4.98×10^{-8}
rs745636	chr15:70457720	G/A	0.0392	0.0122	7.16×10^{-10}
rs74022964	chr15:73677264	T/C	0.1132	0.009	3.51×10^{-36}
rs12908004	chr15:80676925	G/A	0.0732	0.009	4.12×10^{-16}
rs2759301	chr15:80994288	A/G	0.039	0.0067	5.04×10^{-9}
rs4965430	chr15:99268850	C/G	0.0441	0.0069	1.26×10^{-10}
rs118159104	chr16:1676804	G/T	0.1737	0.0325	9.28×10^{-8}
rs140185678	chr16:2003016	A/G	0.1659	0.0218	2.43×10^{-14}
rs77316573	chr16:2265271	T/C	0.0529	0.0089	3.27×10^{-9}
rs2359171	chr16:73053022	A/T	0.1746	0.0086	4.65×10^{-91}
rs876727	chr16:73067761	T/G	0.084	0.0084	1.97×10^{-23}

rs7225165	chr17:1309850	G/A	0.0655	0.0111	3.20×10^{-9}
rs9899183	chr17:7452977	T/C	0.0452	0.0075	2.02×10^{-9}
rs72811294	chr17:12618680	G/C	0.072	0.0106	9.67×10^{-12}
rs11658278	chr17:38031164	T/C	0.0443	0.0067	3.47×10^{-11}
rs1563304	chr17:44874453	T/C	0.0644	0.0092	2.56×10^{-12}
rs12604076	chr17:76773638	T/C	0.0365	0.0066	3.63×10^{-8}
rs9953366	chr18:46474192	C/T	0.049	0.0073	1.82×10^{-11}
rs9963878	chr18:48679522	C/T	0.0653	0.012	4.85×10^{-8}
rs8088085	chr18:48708548	A/C	0.0365	0.0067	4.79×10^{-8}
rs2974231	chr19:48170757	A/G	0.033	0.0104	1.11×10^{-9}
rs2145274	chr20:6572014	A/C	0.0169	0.0231	7.47×10^{-12}
rs2834618	chr21:36119111	T/G	0.0944	0.0112	3.41×10^{-17}
rs56040242	chr21:45766944	A/G	0.0417	0.0121	1.26×10^{-8}
rs464901	chr22:18597502	T/C	0.0508	0.0072	1.53×10^{-12}

rs133902	chr22:26164079	T/C	0.0419	0.0068	9.14×10^{-10}

From: Nielsen JB, Thorolfsdottir RB, Fritsche LG, Zhou W, Skov MW, Graham SE, Herron TJ, McCarthy S, Schmidt EM, Sveinbjornsson G, et al. Biobank-driven genomic discovery yields new insight into atrial fibrillation biology. *Nat Genet*. 2018;50:1234-1239. doi: 10.1038/s41588-018-0171-3

Table S2. Iron-related 20 SNPs

Chr	RsID	Physical. Pos	Reference Allele	Risk Allele	Freq	Info
2	rs744653	1.9×10^8	C	T	0.858289	0.993131
3	rs1799852	1.33×10^8	C	T	0.096052	1
3	rs8177240	1.33×10^8	T	G	0.342583	0.997957
3	rs3811647	1.33×10^8	G	A	0.339334	1
4	rs2245321	1.19×10^8	C	T	0.733547	0.999007
6	rs1799945	26091179	C	G	0.145742	1
6	rs1800562	26093141	G	A	0.073364	0.997254
6	rs3923809	38440970	A	G	0.307114	1
8	rs4921915	18272466	G	A	0.774543	0.999584
9	rs651007	1.36×10^8	C	T	0.204646	1
11	rs6486121	13355770	C	T	0.626766	0.997253

11	rs174577	61604814	C	A	0.350486	0.998255
15	rs1062980	78792527	T	C	0.386898	0.999555
17	rs411988	56709034	G	A	0.546521	0.997588
20	rs235756	6767111	A	G	0.3589	1
22	rs855791	37462936	A	G	0.564611	0.987835
22	rs5756506	37467392	G	C	0.376662	1
22	rs4820268	37469591	G	A	0.538384	1
22	rs2413450	37470224	T	C	0.541599	1
22	rs2235324	37485724	T	C	0.388859	1

Table S3. Association results for the 185 single nucleotide polymorphisms analyzed and the distribution of Atrial fibrillation.

SNP	Risk-Free Genome				Risk Genome			
	Iron Intake Moderate		Iron Intake High		Iron Intake Moderate		Iron Intake High	
	HR (95CI%)	*P*-Value	HR (95CI%)	*p*-Value	HR (95CI%)	*p*-Value	HR (95CI%)	*p*-Value
rs10006327	1.12(0.97,1.28)	0.11	1.13(0.96,1.33)	0.13	1.03(0.95,1.1)	0.52	1.13(1.03,1.23)	0.01
rs10213171	1.05(0.98,1.13)	0.14	1.12(1.03,1.21)	0.01	0.98(0.81,1.19)	0.87	1.23(0.99,1.52)	0.07
rs10458660	1.07(0.98,1.16)	0.11	1.19(1.08,1.30)	<0.001	1.01(0.9,1.13)	0.93	1.01(0.88,1.16)	0.87
rs10465885	1.04(0.91,1.20)	0.56	1.12(0.95,1.32)	0.17	1.05(0.97,1.13)	0.23	1.13(1.04,1.23)	0.01
rs10520260	1.06(0.96,1.16)	0.26	1.12(1.00,1.25)	0.05	1.04(0.95,1.13)	0.44	1.13(1.02,1.26)	0.02
rs10741807	1.22(0.93,1.61)	0.15	1.03(0.73,1.44)	0.89	1.04(0.97,1.11)	0.31	1.13(1.05,1.23)	<0.01
rs10749053	1.10(0.71,1.70)	0.67	1.22(0.74,2.02)	0.43	1.05(0.98,1.12)	0.18	1.13(1.04,1.22)	<0.01
rs10753933	1.05(0.92,1.21)	0.46	1.17(0.99,1.37)	0.06	1.04(0.97,1.12)	0.26	1.12(1.03,1.22)	0.01

rs10760361	1.03(0.93,1.15)	0.54	1.11(0.98,1.25)	0.10	1.05(0.97,1.15)	0.22	1.14(1.03,1.26)	0.01
rs10773657	1.25(0.72,2.16)	0.42	0.87(0.44,1.75)	0.71	1.04(0.98,1.11)	0.21	1.13(1.05,1.22)	<0.01
rs10804493	0.92(0.76,1.11)	0.39	0.97(0.77,1.22)	0.80	1.06(0.99,1.14)	0.08	1.15(1.06,1.25)	<0.01
rs10821415	1.08(0.96,1.21)	0.20	1.09(0.95,1.25)	0.22	1.03(0.95,1.12)	0.45	1.15(1.05,1.26)	<0.01
rs10873298	0.97(0.83,1.14)	0.74	1.01(0.84,1.23)	0.88	1.06(0.99,1.14)	0.10	1.15(1.06,1.25)	<0.01
rs11125871	1.01(0.91,1.13)	0.81	1.13(1.00,1.29)	0.05	1.06(0.98,1.15)	0.14	1.12(1.02,1.24)	0.02
rs11156751	1.07(0.97,1.17)	0.16	1.13(1.01,1.26)	0.03	1.02(0.93,1.12)	0.65	1.13(1.01,1.26)	0.03
rs11264280	0.98(0.89,1.08)	0.67	1.08(0.96,1.22)	0.18	1.10(1.01,1.20)	0.03	1.16(1.05,1.29)	<0.01
rs114904067	1.03(0.97,1.11)	0.33	1.13(1.04,1.22)	<0.01	1.28(0.96,1.71)	0.09	1.16(0.82,1.63)	0.41
rs11590635	1.05(0.98,1.12)	0.17	1.13(1.05,1.22)	<0.01	1.01(0.75,1.36)	0.93	1.11(0.79,1.58)	0.54
rs11598047	1.06(0.98,1.15)	0.14	1.16(1.05,1.27)	<0.01	1.01(0.9,1.140)	0.85	1.07(0.93,1.23)	0.32
rs11614818	0.93(0.77,1.11)	0.41	0.99(0.80,1.24)	0.95	1.06(0.99,1.14)	0.08	1.15(1.06,1.25)	<0.01
rs11658278	1.01(0.89,1.16)	0.83	1.15(0.98,1.34)	0.08	1.06(0.98,1.14)	0.15	1.12(1.03,1.23)	0.01
rs11689011	1.08(0.93,1.26)	0.31	1.15(0.96,1.38)	0.13	1.04(0.97,1.12)	0.31	1.12(1.03,1.22)	0.01

rs116904997	1.06(0.99,1.13)	0.11	1.13(1.05,1.23)	<0.01	0.86(0.63,1.17)	0.33	0.99(0.69,1.42)	0.96
rs11773845	0.93(0.79,1.10)	0.40	0.88(0.72,1.07)	0.21	1.07(0.99,1.15)	0.07	1.18(1.08,1.28)	<0.001
rs117984853	1.04(0.97,1.12)	0.26	1.15(1.06,1.25)	<0.01	1.06(0.91,1.23)	0.48	1.03(0.86,1.24)	0.72
rs118159104	1.04(0.97,1.11)	0.30	1.12(1.04,1.22)	<0.01	1.43(0.96,2.12)	0.08	1.28(0.79,2.07)	0.31
rs12188351	1.02(0.95,1.09)	0.66	1.09(1.01,1.19)	0.03	1.36(1.10,1.68)	<0.01	1.47(1.15,1.87)	<0.01
rs12211255	1.04(0.97,1.12)	0.31	1.14(1.05,1.24)	<0.01	1.07(0.93,1.24)	0.35	1.07(0.90,1.27)	0.44
rs12245149	1.13(0.99,1.28)	0.07	1.23(1.06,1.42)	0.01	1.02(0.94,1.10)	0.67	1.09(1.00,1.19)	0.06
rs12426679	1.12(0.99,1.28)	0.07	1.12(0.96,1.30)	0.15	1.02(0.94,1.10)	0.61	1.13(1.04,1.24)	0.01
rs12604076	1.09(0.95,1.26)	0.21	1.21(1.03,1.43)	0.02	1.03(0.96,1.11)	0.39	1.11(1.01,1.21)	0.02
rs12648245	1.04(0.97,1.11)	0.32	1.11(1.02,1.20)	0.02	1.11(0.92,1.33)	0.26	1.28(1.04,1.57)	0.02
rs12700233	1.09(0.98,1.23)	0.12	1.24(1.09,1.42)	<0.01	1.02(0.94,1.11)	0.57	1.07(0.98,1.18)	0.15
rs1278493	1.13(0.97,1.32)	0.13	1.19(1.00,1.42)	0.05	1.03(0.96,1.11)	0.46	1.12(1.02,1.21)	0.01
rs12809354	1.05(0.97,1.13)	0.25	1.12(1.02,1.23)	0.01	1.04(0.92,1.18)	0.53	1.15(0.99,1.32)	0.07
rs12908004	1.04(0.96,1.12)	0.37	1.10(1.01,1.21)	0.04	1.07(0.95,1.21)	0.27	1.19(1.04,1.37)	0.01

rs13077048	1.04(0.93,1.16)	0.49	1.08(0.94,1.23)	0.28	1.05(0.97,1.14)	0.25	1.15(1.05,1.27)	<0.01
rs13195459	1.07(0.97,1.18)	0.18	1.17(1.04,1.32)	0.01	1.03(0.94,1.12)	0.55	1.10(0.99,1.21)	0.08
rs133902	1.04(0.93,1.17)	0.49	1.06(0.92,1.22)	0.43	1.05(0.97,1.13)	0.24	1.16(1.06,1.27)	<0.01
rs138311480	1.04(0.97,1.11)	0.22	1.14(1.05,1.23)	<0.01	1.12(0.81,1.53)	0.50	0.91(0.62,1.33)	0.62
rs140185678	1.05(0.98,1.13)	0.15	1.14(1.05,1.23)	<0.01	0.99(0.79,1.24)	0.91	1.02(0.79,1.34)	0.86
rs1458038	1.04(0.94,1.14)	0.47	1.13(1.01,1.26)	0.03	1.06(0.96,1.16)	0.25	1.13(1.01,1.26)	0.03
rs146518726	1.05(0.98,1.12)	0.16	1.13(1.04,1.22)	<0.01	0.98(0.72,1.34)	0.92	1.16(0.82,1.66)	0.40
rs147301839	1.05(0.98,1.12)	0.16	1.13(1.05,1.22)	<0.01	0.76(0.39,1.47)	0.41	0.93(0.43,2.00)	0.85
rs1532170	1.11(0.99,1.25)	0.07	1.19(1.04,1.36)	0.01	1.01(0.94,1.10)	0.73	1.10(1.00,1.21)	0.05
rs1545300	1.05(0.96,1.16)	0.28	1.16(1.04,1.29)	0.01	1.04(0.95,1.14)	0.44	1.10(0.99,1.23)	0.08
rs1563304	1.02(0.94,1.10)	0.64	1.10(1.00,1.21)	0.05	1.10(0.98,1.24)	0.10	1.19(1.04,1.36)	0.01
rs17005647	1.04(0.94,1.15)	0.44	1.11(0.98,1.25)	0.10	1.05(0.96,1.14)	0.28	1.14(1.03,1.26)	0.01
rs17118812	1.05(0.95,1.15)	0.34	1.14(1.02,1.27)	0.02	1.05(0.95,1.15)	0.34	1.12(1.00,1.25)	0.04
rs17380837	0.99(0.91,1.09)	0.90	1.07(0.96,1.19)	0.19	1.10(1.00,1.21)	0.04	1.19(1.07,1.33)	<0.01

rs17430364	1.06(0.98, 1.15)	0.16	1.15(1.04, 1.26)	<0.01	1.02(0.91, 1.14)	0.74	1.09(0.96, 1.24)	0.19
rs1957021	1.07(0.98, 1.17)	0.13	1.13(1.02, 1.25)	0.02	1.02(0.92, 1.12)	0.75	1.13(1.01, 1.27)	0.04
rs1980728	0.88(0.67, 1.14)	0.32	1.01(0.75, 1.37)	0.94	1.06(0.99, 1.13)	0.10	1.14(1.05, 1.23)	<0.01
rs2012809	1.18(0.77, 1.80)	0.44	1.53(0.95, 2.47)	0.08	1.04(0.98, 1.12)	0.21	1.12(1.03, 1.21)	0.01
rs2031522	1.00(0.90, 1.11)	0.96	1.07(0.94, 1.20)	0.31	1.08(0.99, 1.18)	0.08	1.17(1.06, 1.29)	<0.01
rs2040862	1.06(0.98, 1.15)	0.18	1.14(1.03, 1.25)	0.01	1.02(0.92, 1.15)	0.68	1.11(0.98, 1.27)	0.11
rs2145274	1.04(0.97, 1.11)	0.30	1.13(1.04, 1.22)	0.01	1.09(0.92, 1.30)	0.32	1.14(0.93, 1.40)	0.21
rs2274115	0.96(0.77, 1.2)	0.73	1.03(0.79, 1.34)	0.84	1.05(0.98, 1.13)	0.13	1.14(1.05, 1.23)	<0.01
rs2283038	1.00(0.92, 1.09)	0.96	1.08(0.98, 1.19)	0.13	1.11(1.00, 1.23)	0.04	1.20(1.07, 1.35)	<0.01
rs2288327	1.04(0.96, 1.12)	0.38	1.09(1.00, 1.20)	0.06	1.07(0.95, 1.20)	0.28	1.21(1.05, 1.38)	0.01
rs2291437	1.06(0.99, 1.15)	0.10	1.12(1.03, 1.23)	0.01	0.98(0.85, 1.13)	0.81	1.14(0.97, 1.34)	0.10
rs2308655	1.08(0.98, 1.2)	0.13	1.24(1.10, 1.40)	<0.001	1.02(0.94, 1.11)	0.60	1.05(0.95, 1.16)	0.32
rs2359171	1.08(0.99, 1.17)	0.07	1.17(1.07, 1.29)	<0.01	0.99(0.89, 1.11)	0.88	1.05(0.93, 1.20)	0.42
rs244017	1.01(0.75, 1.37)	0.94	1.12(0.78, 1.59)	0.54	1.05(0.98, 1.12)	0.17	1.13(1.04, 1.22)	<0.01

rs2540949	1.06(0.95,1.17)	0.29	1.17(1.04,1.32)	0.01	1.04(0.95,1.13)	0.39	1.10(0.99,1.21)	0.07
rs2738413	1.06(0.93,1.21)	0.39	1.12(0.96,1.3)	0.16	1.04(0.97,1.13)	0.28	1.13(1.04,1.24)	0.01
rs2759301	1.02(0.90,1.14)	0.78	1.11(0.97,1.27)	0.14	1.06(0.98,1.15)	0.16	1.14(1.04,1.25)	0.01
rs2834618	1.05(0.98,1.13)	0.18	1.10(1.02,1.20)	0.02	1.02(0.87,1.20)	0.79	1.24(1.04,1.49)	0.02
rs28387148	1.06(0.98,1.14)	0.13	1.15(1.05,1.25)	<0.01	1.00(0.87,1.16)	1.00	1.06(0.89,1.25)	0.53
rs284277	0.93(0.79,1.11)	0.43	0.98(0.80,1.19)	0.81	1.07(0.99,1.15)	0.07	1.16(1.06,1.26)	<0.01
rs28439930	1.14(1.00,1.29)	0.04	1.20(1.04,1.39)	0.01	1.01(0.94,1.09)	0.76	1.10(1.01,1.20)	0.04
rs2860482	1.12(0.89,1.42)	0.34	1.02(0.76,1.35)	0.91	1.04(0.97,1.11)	0.27	1.14(1.05,1.23)	<0.01
rs2885697	0.99(0.81,1.20)	0.91	1.12(0.88,1.41)	0.36	1.05(0.98,1.13)	0.14	1.13(1.04,1.23)	<0.01
rs2974231	1.03(0.88,1.20)	0.69	1.02(0.85,1.22)	0.85	1.05(0.98,1.13)	0.20	1.15(1.06,1.26)	<0.01
rs3176326	1.05(0.97,1.14)	0.23	1.14(1.03,1.25)	0.01	1.04(0.93,1.16)	0.49	1.12(0.98,1.27)	0.10
rs337705	1.02(0.92,1.14)	0.70	1.12(0.99,1.27)	0.08	1.06(0.98,1.15)	0.17	1.13(1.03,1.25)	0.01
rs34080181	1.05(0.95,1.17)	0.36	1.10(0.97,1.25)	0.12	1.04(0.96,1.13)	0.33	1.14(1.04,1.26)	0.01
rs34104130	1.01(0.92,1.10)	0.89	1.11(1.00,1.23)	0.05	1.10(0.99,1.21)	0.06	1.15(1.03,1.29)	0.02

rs34969716	0.99(0.90,1.09)	0.80	1.09(0.97,1.22)	0.14	1.10(1.00,1.20)	0.04	1.17(1.05,1.30)	<0.01
rs35005436	1.09(1.01,1.18)	0.03	1.18(1.07,1.29)	<0.01	0.96(0.85,1.07)	0.44	1.03(0.90,1.18)	0.70
rs35006907	1.06(0.96,1.17)	0.25	1.15(1.03,1.29)	0.02	1.04(0.95,1.13)	0.44	1.11(1.00,1.23)	0.05
rs35176054	1.03(0.95,1.11)	0.48	1.10(1.00,1.20)	0.04	1.11(0.97,1.27)	0.15	1.23(1.05,1.44)	0.01
rs35544454	1.06(0.98,1.15)	0.13	1.12(1.02,1.23)	0.02	1.01(0.9,1.13)	0.86	1.14(1.00,1.30)	0.06
rs35569628	1.07(0.98,1.16)	0.12	1.16(1.05,1.28)	<0.01	1.01(0.91,1.12)	0.81	1.08(0.95,1.22)	0.23
rs35620480	1.05(0.97,1.14)	0.21	1.12(1.02,1.22)	0.02	1.04(0.92,1.17)	0.57	1.16(1.01,1.34)	0.03
rs3820888	1.02(0.92,1.14)	0.66	1.08(0.95,1.23)	0.23	1.06(0.98,1.15)	0.17	1.16(1.05,1.27)	<0.01
rs3853445	1.08(0.99,1.18)	0.09	1.11(1.00,1.23)	0.05	1.00(0.91,1.11)	0.93	1.15(1.03,1.30)	0.02
rs3951016	1.06(0.93,1.20)	0.39	1.21(1.05,1.40)	0.01	1.04(0.97,1.13)	0.29	1.10(1.00,1.20)	0.04
rs4073778	1.14(0.97,1.33)	0.10	1.12(0.93,1.35)	0.22	1.03(0.96,1.1)	0.47	1.13(1.04,1.23)	0.01
rs422068	1.07(0.96,1.19)	0.21	1.14(1.01,1.29)	0.03	1.03(0.95,1.12)	0.48	1.12(1.01,1.23)	0.03
rs464901	1.06(0.96,1.17)	0.25	1.12(1.00,1.26)	0.05	1.04(0.95,1.13)	0.43	1.13(1.02,1.26)	0.02
rs4743034	1.07(0.98,1.17)	0.12	1.21(1.10,1.34)	<0.001	1.01(0.91,1.12)	0.81	1.01(0.90,1.14)	0.83

rs4871397	0.63(0.25,1.58)	0.32	1.09(0.37,3.23)	0.87	1.05(0.98,1.12)	0.14	1.13(1.05,1.22)	<0.01
rs4896104	1.17(1.01,1.37)	0.04	1.31(1.10,1.56)	<0.01	1.02(0.95,1.09)	0.64	1.09(1.00,1.19)	0.05
rs4935786	0.98(0.79,1.23)	0.88	0.98(0.75,1.27)	0.85	1.05(0.98,1.13)	0.15	1.14(1.06,1.24)	<0.01
rs4951258	1.10(0.98,1.22)	0.10	1.11(0.98,1.27)	0.10	1.02(0.94,1.11)	0.65	1.14(1.03,1.25)	0.01
rs4963776	1.02(0.94,1.10)	0.60	1.14(1.04,1.25)	<0.01	1.10(0.98,1.24)	0.11	1.09(0.95,1.26)	0.24
rs4965430	1.17(0.98,1.38)	0.08	1.17(0.96,1.42)	0.13	1.03(0.95,1.10)	0.49	1.12(1.03,1.22)	0.01
rs4999127	1.41(0.81,2.45)	0.22	1.32(0.70,2.47)	0.39	1.04(0.98,1.11)	0.22	1.13(1.04,1.22)	<0.01
rs55693294	1.05(0.98,1.13)	0.15	1.14(1.05,1.24)	<0.01	1.00(0.83,1.20)	0.98	1.06(0.85,1.32)	0.60
rs55734480	1.06(0.97,1.16)	0.21	1.15(1.03,1.28)	0.01	1.03(0.94,1.13)	0.52	1.11(0.99,1.24)	0.07
rs55985730	1.08(1.00,1.16)	0.04	1.15(1.06,1.25)	<0.01	0.84(0.70,1.01)	0.07	0.99(0.80,1.22)	0.91
rs56040242	1.08(0.99,1.18)	0.08	1.07(0.96,1.18)	0.22	1.00(0.90,1.11)	1.00	1.23(1.09,1.38)	<0.01
rs56181519	1.06(0.97,1.15)	0.22	1.11(1.00,1.23)	0.05	1.03(0.93,1.14)	0.54	1.16(1.03,1.30)	0.02
rs56201652	1.08(0.99,1.18)	0.08	1.09(0.98,1.21)	0.10	1.00(0.91,1.11)	0.96	1.17(1.05,1.31)	0.01
rs565449	1.18(0.96,1.44)	0.11	1.03(0.81,1.32)	0.80	1.03(0.96,1.1)	0.40	1.14(1.05,1.23)	<0.01

rs577676	1.10(0.98,1.24)	0.10	1.09(0.95,1.25)	0.20	1.02(0.94,1.10)	0.65	1.15(1.04,1.26)	<0.01
rs60212594	1.04(0.96,1.12)	0.35	1.13(1.03,1.23)	0.01	1.07(0.94,1.23)	0.30	1.13(0.97,1.32)	0.12
rs60902112	1.04(0.95,1.13)	0.36	1.19(1.08,1.31)	<0.01	1.05(0.95,1.17)	0.30	1.05(0.93,1.18)	0.45
rs61501369	1.04(0.95,1.13)	0.41	1.13(1.02,1.26)	0.02	1.05(0.96,1.16)	0.28	1.12(1.00,1.26)	0.05
rs62274627	1.03(0.93,1.13)	0.61	1.15(1.03,1.29)	0.01	1.06(0.97,1.16)	0.19	1.11(1.00,1.23)	0.06
rs62521286	1.07(0.99,1.14)	0.08	1.16(1.06,1.26)	<0.01	0.93(0.78,1.11)	0.42	0.97(0.79,1.19)	0.79
rs6462079	1.22(0.92,1.60)	0.16	1.17(0.85,1.61)	0.35	1.04(0.97,1.11)	0.30	1.13(1.04,1.22)	<0.01
rs6560886	0.95(0.68,1.31)	0.74	0.82(0.55,1.23)	0.34	1.05(0.98,1.12)	0.14	1.14(1.06,1.24)	<0.01
rs6580277	1.08(0.99,1.18)	0.08	1.19(1.07,1.31)	<0.01	1.00(0.91,1.11)	0.98	1.06(0.94,1.19)	0.35
rs6596717	1.07(0.90,1.26)	0.45	1.23(1.02,1.50)	0.03	1.04(0.97,1.12)	0.25	1.11(1.02,1.21)	0.01
rs6689306	1.08(0.92,1.26)	0.34	1.06(0.88,1.27)	0.55	1.04(0.97,1.12)	0.30	1.14(1.05,1.24)	<0.01
rs67249485	1.00(0.92,1.09)	0.98	1.12(1.02,1.24)	0.02	1.11(1.01,1.23)	0.04	1.13(1.01,1.28)	0.04
rs6747542	1.02(0.91,1.15)	0.74	1.1(0.95,1.26)	0.19	1.06(0.98,1.15)	0.15	1.14(1.04,1.25)	<0.01
rs6790396	1.17(0.99,1.38)	0.07	1.15(0.94,1.40)	0.18	1.02(0.95,1.10)	0.52	1.13(1.04,1.22)	0.01

rs67969609	1.05(0.97,1.12)	0.21	1.14(1.05,1.23)	<0.01	1.03(0.87,1.23)	0.69	1.08(0.88,1.32)	0.46
rs6829664	1.03(0.94,1.13)	0.49	1.14(1.03,1.26)	0.01	1.07(0.97,1.17)	0.20	1.12(1.00,1.25)	0.06
rs6841049	1.04(0.9,1.19)	0.62	1.04(0.88,1.22)	0.67	1.05(0.98,1.13)	0.19	1.15(1.06,1.26)	<0.01
rs6850025	1.05(0.98,1.13)	0.16	1.16(1.07,1.26)	<0.001	1.01(0.83,1.22)	0.94	0.88(0.70,1.11)	0.27
rs6891790	1.06(0.97,1.16)	0.20	1.11(1.00,1.23)	0.06	1.03(0.93,1.13)	0.58	1.15(1.03,1.28)	0.02
rs6994744	1.00(0.87,1.14)	0.96	1.08(0.92,1.25)	0.35	1.06(0.99,1.15)	0.12	1.15(1.05,1.25)	<0.01
rs7096385	1.35(0.43,4.21)	0.60	1.05(0.28,3.93)	0.94	1.04(0.98,1.12)	0.19	1.13(1.04,1.22)	<0.01
rs7134121	1.11(1.01,1.23)	0.04	1.16(1.04,1.31)	0.01	1.00(0.91,1.09)	0.96	1.10(0.99,1.22)	0.06
rs71454237	1.07(0.99,1.17)	0.10	1.16(1.06,1.28)	<0.01	1.00(0.90,1.11)	0.98	1.07(0.94,1.21)	0.31
rs7170477	1.04(0.95,1.14)	0.43	1.10(0.99,1.23)	0.08	1.05(0.96,1.16)	0.26	1.15(1.03,1.28)	0.01
rs7225165	1.05(0.97,1.13)	0.23	1.11(1.02,1.21)	0.01	1.05(0.90,1.21)	0.54	1.19(1.01,1.41)	0.04
rs72700114	1.07(0.99,1.15)	0.08	1.14(1.05,1.24)	<0.01	0.94(0.80,1.10)	0.46	1.09(0.91,1.31)	0.37
rs72700118	1.05(0.97,1.13)	0.25	1.16(1.06,1.26)	<0.01	1.05(0.92,1.20)	0.46	1.04(0.89,1.21)	0.61
rs72721963	1.02(0.95,1.1)	0.54	1.11(1.02,1.20)	0.02	1.16(0.99,1.35)	0.07	1.23(1.03,1.47)	0.02

rs72811294	1.05(0.98,1.13)	0.18	1.12(1.03,1.22)	0.01	1.03(0.89,1.19)	0.72	1.17(0.98,1.38)	0.08
rs72926475	1.06(0.98,1.14)	0.15	1.14(1.04,1.24)	<0.01	1.01(0.88,1.16)	0.86	1.09(0.93,1.28)	0.26
rs73041705	0.99(0.9,1.09)	0.84	1.09(0.98,1.22)	0.11	1.1(1.01,1.21)	0.04	1.17(1.05,1.30)	0.01
rs73241997	1.02(0.95,1.1)	0.59	1.11(1.01,1.22)	0.02	1.11(0.98,1.26)	0.09	1.18(1.02,1.36)	0.03
rs73366713	1.07(0.99,1.16)	0.08	1.17(1.07,1.28)	<0.01	0.98(0.86,1.11)	0.71	1.02(0.87,1.19)	0.81
rs7373065	57445.16(0, Inf)	1.00	0(0, Inf)	1.00	1.05(0.98,1.12)	0.18	1.13(1.05,1.22)	<0.01
rs7374540	1.01(0.86,1.19)	0.90	1.14(0.94,1.38)	0.20	1.05(0.98,1.13)	0.16	1.13(1.04,1.23)	0.01
rs74022964	1.03(0.96,1.12)	0.41	1.12(1.02,1.23)	0.02	1.07(0.95,1.21)	0.24	1.15(1.00,1.33)	0.04
rs745636	1.04(0.95,1.13)	0.42	1.09(0.99,1.20)	0.10	1.06(0.96,1.18)	0.26	1.19(1.05,1.34)	0.01
rs74884082	1.03(0.94,1.12)	0.54	1.10(0.99,1.21)	0.07	1.07(0.97,1.19)	0.19	1.17(1.04,1.32)	0.01
rs7508	1.12(0.88,1.43)	0.34	1.11(0.83,1.48)	0.47	1.04(0.97,1.11)	0.26	1.13(1.04,1.22)	<0.01
rs7529220	0.94(0.56,1.58)	0.82	1.25(0.70,2.23)	0.46	1.05(0.98,1.12)	0.17	1.13(1.04,1.22)	<0.01
rs7578393	1.38(0.92,2.06)	0.12	1.68(1.06,2.67)	0.03	1.04(0.97,1.11)	0.28	1.11(1.03,1.20)	0.01

rs76097649	1.06(0.99,1.14)	0.10	1.17(1.08,1.28)	<0.001	0.98(0.84,1.14)	0.75	0.94(0.78,1.12)	0.48
rs7612445	1.04(0.96,1.13)	0.35	1.14(1.04,1.26)	0.01	1.06(0.95,1.18)	0.32	1.10(0.97,1.26)	0.14
rs7650482	0.97(0.80,1.16)	0.71	0.97(0.78,1.21)	0.79	1.06(0.99,1.14)	0.11	1.15(1.06,1.25)	<0.01
rs7687819	1.05(0.96,1.14)	0.27	1.14(1.03,1.26)	0.01	1.04(0.94,1.16)	0.45	1.11(0.98,1.25)	0.11
rs77316573	1.02(0.94,1.10)	0.71	1.14(1.03,1.25)	0.01	1.11(0.99,1.24)	0.07	1.11(0.98,1.27)	0.11
rs775498	1.06(0.96,1.16)	0.26	1.12(1.00,1.25)	0.04	1.04(0.94,1.14)	0.46	1.14(1.02,1.27)	0.02
rs7789146	1.02(0.94,1.10)	0.69	1.11(1.01,1.22)	0.03	1.12(0.99,1.26)	0.07	1.17(1.02,1.34)	0.03
rs7834729	1.04(0.97,1.12)	0.28	1.12(1.03,1.23)	0.01	1.06(0.92,1.22)	0.44	1.14(0.96,1.35)	0.14
rs79187193	1.03(0.96,1.10)	0.48	1.11(1.03,1.20)	0.01	1.30(1.03,1.65)	0.03	1.34(1.02,1.75)	0.04
rs79399769	1.06(0.99,1.13)	0.11	1.14(1.05,1.23)	<0.01	0.87(0.66,1.15)	0.33	0.94(0.68,1.30)	0.70
rs8088085	1.13(1.01,1.28)	0.04	1.18(1.03,1.36)	0.02	1.01(0.93,1.09)	0.81	1.11(1.01,1.21)	0.03
rs876727	1.04(0.96,1.13)	0.33	1.13(1.03,1.24)	0.01	1.05(0.95,1.18)	0.34	1.13(0.99,1.28)	0.07
rs883079	1.07(0.83,1.37)	0.61	1.11(0.83,1.50)	0.47	1.04(0.98,1.12)	0.21	1.13(1.04,1.22)	<0.01
rs9401451	1.06(0.99,1.14)	0.09	1.14(1.05,1.24)	<0.01	0.97(0.83,1.13)	0.67	1.09(0.91,1.30)	0.35

rs9506925	0.99(0.90,1.08)	0.80	1.06(0.95,1.18)	0.29	1.11(1.01,1.22)	0.03	1.21(1.08,1.35)	<0.01
rs9899183	0.91(0.71,1.16)	0.45	1.13(0.85,1.50)	0.41	1.06(0.99,1.13)	0.10	1.13(1.04,1.22)	<0.01
rs9953366	0.96(0.78,1.17)	0.67	1.05(0.83,1.33)	0.69	1.06(0.99,1.13)	0.12	1.14(1.05,1.23)	<0.01
rs9963878	1.05(0.98,1.13)	0.15	1.13(1.04,1.23)	0.01	1.01(0.86,1.18)	0.91	1.13(0.94,1.36)	0.18
rs855791	0.93(0.80,1.07)	0.31	1.16(0.98,1.37)	0.09	1.08(1.00,1.16)	0.05	1.12(1.03,1.22)	0.01
rs1062980	1.10(0.98,1.22)	0.10	1.15(1.01,1.30)	0.03	1.02(0.94,1.1)	0.70	1.12(1.01,1.23)	0.03
rs8177240	1.08(0.97,1.19)	0.15	1.17(1.04,1.32)	0.01	1.02(0.94,1.12)	0.59	1.10(0.99,1.21)	0.08
rs744653	1.34(0.80,2.26)	0.27	1.49(0.83,2.66)	0.18	1.04(0.98,1.11)	0.23	1.12(1.04,1.21)	<0.01
rs6486121	1.02(0.85,1.21)	0.86	1.05(0.85,1.29)	0.65	1.05(0.98,1.13)	0.17	1.14(1.05,1.24)	<0.01
rs651007	1.02(0.94,1.11)	0.58	1.09(0.99,1.20)	0.08	1.08(0.97,1.21)	0.14	1.19(1.05,1.35)	0.01
rs1799852	1.03(0.96,1.10)	0.45	1.11(1.02,1.21)	0.02	1.14(0.97,1.33)	0.11	1.22(1.02,1.47)	0.03
rs1799945	1.03(0.95,1.11)	0.47	1.08(0.99,1.18)	0.09	1.1(0.97,1.25)	0.15	1.27(1.10,1.47)	<0.01
rs2235324	1.04(0.93,1.15)	0.51	1.15(1.01,1.30)	0.03	1.05(0.97,1.14)	0.23	1.12(1.01,1.23)	0.03
rs2245321	1.33(1.02,1.72)	0.03	1.25(0.92,1.69)	0.15	1.03(0.96,1.10)	0.43	1.12(1.03,1.21)	0.01

rs235756	1.07(0.96,1.18)	0.21	1.13(1.00,1.28)	0.04	1.03(0.95,1.12)	0.47	1.13(1.02,1.24)	0.02
rs3811647	1.08(0.97,1.19)	0.15	1.16(1.04,1.31)	0.01	1.02(0.94,1.12)	0.60	1.10(0.99,1.22)	0.07
rs3923809	1.12(1.02,1.23)	0.02	1.19(1.06,1.33)	<0.01	0.98(0.90,1.08)	0.71	1.08(0.97,1.19)	0.18
rs411988	1.03(0.89,1.19)	0.72	0.97(0.81,1.15)	0.73	1.05(0.98,1.13)	0.18	1.17(1.07,1.27)	<0.001
rs4921915	1.20(0.88,1.63)	0.25	1.24(0.86,1.77)	0.25	1.04(0.97,1.11)	0.26	1.12(1.04,1.22)	<0.01
rs4820268	0.92(0.80,1.05)	0.21	1.08(0.92,1.27)	0.35	1.09(1.01,1.17)	0.03	1.15(1.05,1.25)	<0.01
rs2413450	0.92(0.80,1.06)	0.24	1.09(0.93,1.28)	0.31	1.08(1.01,1.17)	0.03	1.14(1.05,1.25)	<0.01
rs174577	1.01(0.92,1.12)	0.81	1.17(1.04,1.31)	0.01	1.07(0.98,1.17)	0.12	1.10(0.99,1.22)	0.07
rs1800562	1.05(0.98,1.13)	0.19	1.13(1.04,1.22)	0.01	1.03(0.87,1.22)	0.74	1.14(0.93,1.39)	0.20
rs5756506	0.99(0.89,1.09)	0.80	1.11(0.98,1.25)	0.09	1.09(1.00,1.19)	0.05	1.14(1.04,1.26)	0.01

HR, hazard ratio; CI, confidence interval.

Adjusted for age, sex, ethnicity, smoking, drinking, BMI, diabetes history, antilipemic, iron supplement, hypertension, myocardial infarction, heart failure, stroke.

Table S4. Total number of enrichment of the input genes in FUMA.

Category	Gene Set	N Genes	N Overlap	P	Adj P	Genes	Link
GO_bp	GOBP_TRANSMEMBRANE_RECEPTOR_PROTEIN_SERINE_THREONINE_KINASE_SIGNALING_PATHWAY	376	10	1.88×10^{-8}	0.000146	TF: HFE: CAV2: CAV1: PTK2: SIRT1: SORL1: PXN: SMAD7: TMPRSS6	http://www.gsea-msigdb.org/gsea/msigdb/human/geneset/GOBP_TRANSMEMBRANE_RECEPTOR_PROTEIN_SERINE_THREONINE_KINASE_SIGNALING_PATHWAY
GO_bp	GOBP_HEART_PROCESS	247	8	1.26×10^{-7}	0.000974	TTN: THRB: SCN5A: SCN10A: CAV1: TBX5: SGCG: SMAD7	http://www.gsea-msigdb.org/gsea/msigdb/human/geneset/GOBP_HEART_PROCESS

GO_bp	GOBP_MUSCLE_TISSUE_DEVELOPMENT	410	9	5.20×10^{-7}	0.004031	TTN: SCN5A: CAV2: CAV1: NEURL1: TBX5: SGCG: SMAD7:MYO18B	http://www.gsea-msigdb.org/gsea/msigdb/human/geneset/GOBP_MUSCLE_TISSUE_DEVELOPMENT
GO_bp	GOBP_MEMBRANE_DEPOLARIZATION	74	5	9.22×10^{-7}	0.007146	SCN5A: SCN10A: CAV1: TBX5:SMAD7	http://www.gsea-msigdb.org/gsea/msigdb/human/geneset/GOBP_MEMBRANE_DEPOLARIZATION
GO_bp	GOBP_REGULATION_OF_ATRIAL_CARDIAC_MUSCLE_CELL_MEMBRANE_DEPOLARIZATION	9	3	1.07×10^{-6}	0.008313	SCN5A: SCN10A: TBX5	http://www.gsea-msigdb.org/gsea/msigdb/human/geneset/GOBP_REGULATION_OF_ATRIAL_CARDIAC_MUSCLE_CELL_MEMBRANE_DEPOLARIZATION

GO_bp	GOBP_HEART_DEVELOPMENT	584	10	1.10×10^{-6}	0.008522	TTN: SCN5A: PITX2: HAND2: PTK2: RBM20: TBX5: SGCG: SMAD7: MYO18B	http://www.gsea-msigdb.org/gsea/msigdb/human/geneset/GOBP_HEART_DEVELOPMENT
GWAScatalog	Atrial fibrillation	217	47	4.95×10^{-88}	2.19E-84	CASZ1: HSPG2: SCMH1: KCNN3: LINC01142: ANXA4: GMCL1: GYPC: TEX41: FKBP7: TTN-AS1: TTN: SPATS2L: CAND2: THRB: SCN5A: SCN10A:	

FRMD4B: PPP2R3A:

PITX2: HAND2:

HAND2-AS1:

KDM1B: DEK:

MIR548N: CDK6:

CAV2: CAV1:

FBXO32: PTK2:

LHX3: SIRT1:

MYPN: NEURL1:

RBM20: NAV2:

SORL1: SSPN:

NACA: TBX5:

HIP1R: FBRSL1:

SGCG: IRF2BPL:

GWAScatalog	PR interval	215	16	4.61×10^{-20}	2.04E-16	WNT3: SMAD7: MYO18B HSPG2: TEX41: TTN: THRB: SCN5A: SCN10A: FRMD4B: HAND2: DEK: CAV2: CAV1: PTK2: NAV2: SSPN: TBX5: LRCH1
GWAScatalog	Electrocardiogram morphology (amplitude at temporal datapoints)	381	18	5.57×10^{-19}	2.46E-15	CASZ1: TTN: SPATS2L: CAND2: THRB: SCN5A: SCN10A: PPP2R3A:

						PITX2: HAND2: CAV1: FBXO32: RBM20: NAV2: TBX5: LRCH1: SMAD7: MYO18B
GWAScatalog	Prevalent atrial fibrillation	10	6	2.95×10^{-14}	1.31E-10	KCNN3: ANXA4: GMCL1: PITX2: CAV1: NEURL1
GWAScatalog	P wave duration	16	6	1.11×10^{-12}	4.92E-09	CAND2: SCN5A: SCN10A: CAV2: CAV1:TBX5
GWAScatalog	Red blood cell count	509	13	1.71×10^{-10}	7.58E-07	CASZ1: TEX41: THRB: TF: LIN54: HFE: HLA-B:

GWAScatalog	Electrocardiographic traits (multivariate)	54	6	3.35×10^{-9}	1.48E-05	CDK6: LHX3: FBRSL1: WNT3: SMAD7: TMPRSS6 TTN: SCN5A: SCN10A: PITX2: CAV1: TBX5
GWAScatalog	PR segment duration	10	4	6.07×10^{-9}	2.68E-05	SCN5A: SCN10A: CAV1: LRCH1
GWAScatalog	Ischemic stroke (cardioembolic)	11	4	9.52×10^{-9}	4.21E-05	PITX2: CAV2: CAV1: NEURL1
GWAScatalog	Brugada syndrome	16	4	5.20×10^{-8}	0.00023	SCN5A: SCN10A: TBX5: MYO18B
GWAScatalog	Electrocardiographic traits	17	4	6.79×10^{-8}	0.0003	SCN5A: SCN10A CAV1: TBX5

GWAScatalog	Incident atrial fibrillation	5	3	1.29×10^{-7}	0.000569	KCNN3: PITX2: NEURL1
GWAScatalog	QRS duration	55	5	2.06×10^{-7}	0.000912	SCN5A: SCN10A: CAV1: NACA: TBX5
GWAScatalog	Early onset atrial fibrillation	6	3	2.57×10^{-7}	0.001136	PITX2: HAND2: NEURL1
GWAScatalog	Sick sinus syndrome	7	3	4.48×10^{-7}	0.001984	TTN: SCN10A: PITX2
GWAScatalog	Iron status biomarkers (transferrin levels)	8	3	7.16×10^{-7}	0.003169	TF: HFE: TMPRSS6
GWAScatalog	Systolic blood pressure	704	11	7.35×10^{-7}	0.003253	CASZ1: GYPC: SPATS2L: PPP2R3A: LIN54: HFE: HLA-B:

						CDK6: TBX5: FBRSL1: LRCH1	
GWAScatalog	Hepcidin levels	9	3	1.07×10^{-6}	0.004746	TF: HFE: TMPRSS6	
GWAScatalog	Intraocular pressure	347	8	1.64×10^{-6}	0.007259	TEX41: PPP2R3A: PITX2: CAV2: CAV1: FBXO32: NEURL1: WNT3	
GO_cc	GOCC_PLASMA_MEMBRANE_PROTEIN_COMPLEX	551	10	6.50×10^{-7}	0.000655	HSPG2: SCN5A: SCN10A: TF: HFE: HLA-B: CAV2: CAV1: SSPN: SGCG	http://www.gsea-msigdb.org/gsea/msigdb/human/geneset/GOCC_PLASMA_MEMBRANE_PROTEIN_COMPLEX

Wikipathways	WP_HFE_EFFECT_ON_HEPCIDIN_PRODUCTION	7	3	4.48×10^{-7}	0.000329	HFE: SMAD7: TMPRSS6	http://www.gsea-msigdb.org/gsea/msigdb/human/geneset/WP_HFE_EFFECT_ON_HEPCIDIN_PRODUCTION
Wikipathways	WP_INTEGRINMEDIATED_CELL_ADHESION	98	5	3.72×10^{-6}	0.002727	CAV2: CAV1: PTK2: MYPN: PXN	http://www.gsea-msigdb.org/gsea/msigdb/human/geneset/WP_INTEGRINMEDIATED_CELL_ADHESION
Curated_gene_sets	WP_HFE_EFFECT_ON_HEPCIDIN_PRODUCTION	7	3	4.48×10^{-7}	0.002912	HFE: SMAD7: TMPRSS6	http://www.gsea-msigdb.org/gsea/msigdb/human/geneset/WP_HFE_EFFECT_ON_HEPCIDIN_PRODUCTION
microRNA_targets	MIR4324	263	7	3.01×10^{-6}	0.007828	KCNN3: GMCL1: CAND2: THRB: PTK2: RBM20: PXN	http://www.gsea-msigdb.org/gsea/msigdb/human/geneset/MIR4324
TF_targets	AP4_01	258	7	2.65×10^{-6}	0.00296	PITX2: CDK6: PTK2: MYPN:	http://www.gsea-msigdb.org/gsea/msigdb/human/geneset/AP4_01

TF_targets	CAGCTG_AP4_Q5	1479	14	7.25×10^{-6}	0.008084	SGCG: SMAD7: GLTSCR1 KCNN3: SCN5A: PPP2R3A: PTK2: LHX3: MYPN: NEURL1: NACA: HIP1R: SGCG: LRCH1: IRF2BPL: GLTSCR1:MYO18B	http://www.gsea-msigdb.org/gsea/msigdb/human/geneset/CAGCTG_AP4_Q5
Canonical_Pathways	WP_HFE_EFFECT_ON_HEPCIDIN_PRODUCTION	7	3	4.48×10^{-7}	0.001386	HFE: SMAD7: TMPRSS6	http://www.gsea-msigdb.org/gsea/msigdb/human/geneset/WP_HFE_EFFECT_ON_HEPCIDIN_PRODUCTION

Figure S1. Subgroup analysis

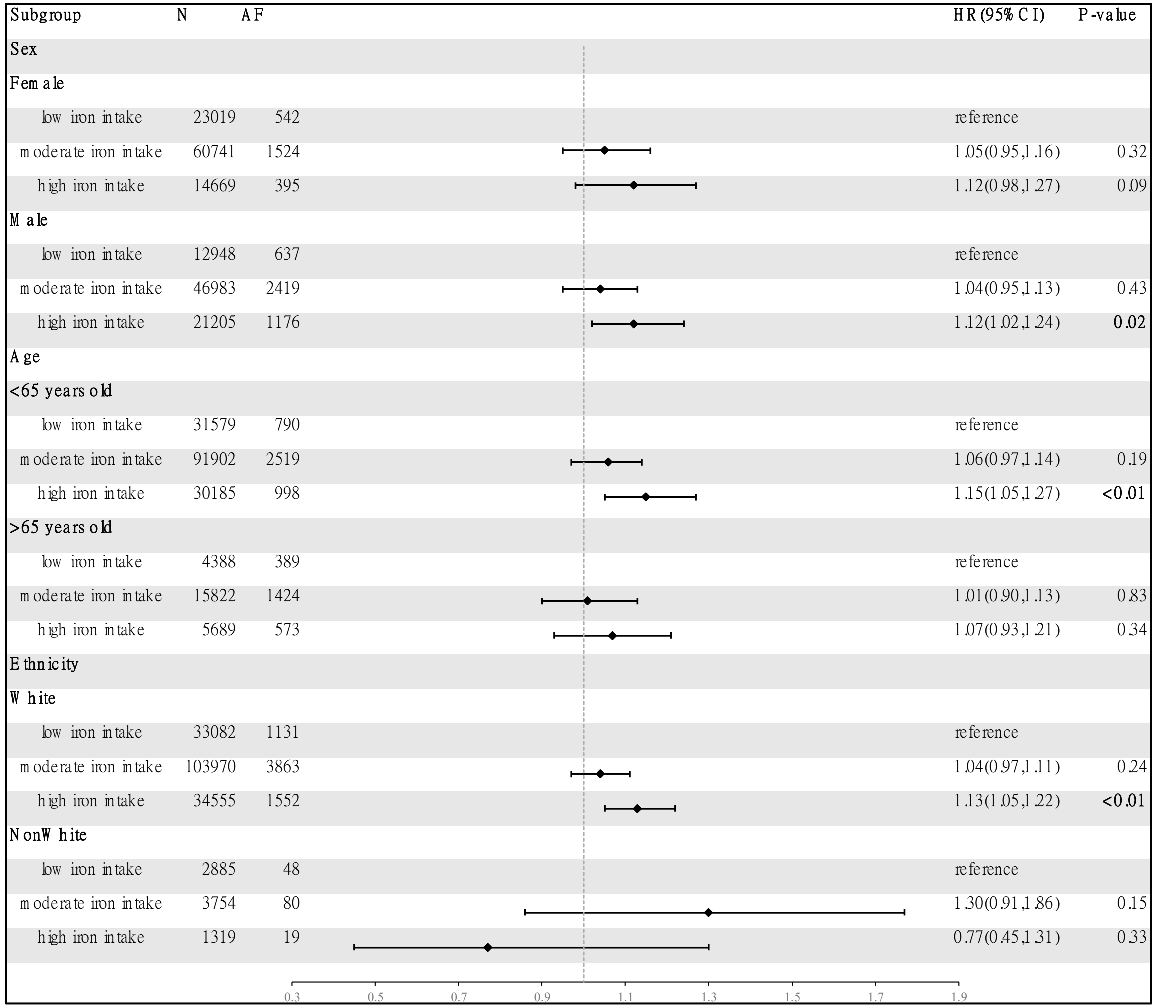

The magnitude of the association between dietary iron intake and the AF risk was greater in White (*p*-value <0.001),

in male participants (P-value=0.02), in younger than 65 years old participants (P-value <0.001).

Covariates included age, sex, ethnicity, smoking, drinking, BMI, diabetes history, antilipemic, iron supplement, hypertension, myocardial infarction, heart failure, stroke.
